# Enhancing the NIR Photocurrent in Single GaAs Nanowires with Radial p-i-n Junctions by Uniaxial Strain

**DOI:** 10.1021/acs.nanolett.1c02468

**Published:** 2021-10-27

**Authors:** Jonatan Holmér, Lunjie Zeng, Thomas Kanne, Peter Krogstrup, Jesper Nygård, Eva Olsson

**Affiliations:** † Department of Physics, Chalmers University of Technology, 412 96 Gothenburg, Sweden; ‡ Center for Quantum Devices, Niels Bohr Institute, University of Copenhagen, Universitetsparken 5, 2100 Copenhagen, Denmark

**Keywords:** II*I*−*V* nanowires, solar cells, strain, *I*−*V* characteristics, photocurrent, EBIC

## Abstract

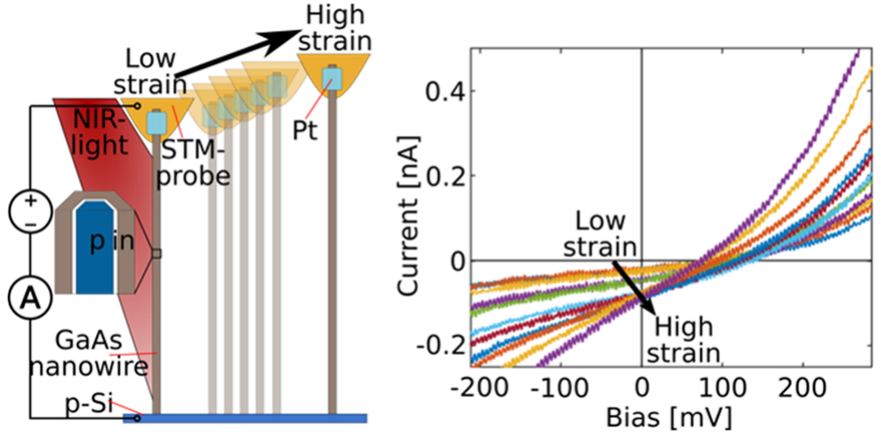

III–V compound nanowires have electrical and optical properties suitable for a wide range of applications, including photovoltaics and photodetectors. Furthermore, their elastic nature allows the use of strain engineering to enhance their performance. Here we have investigated the effect of mechanical strain on the photocurrent and the electrical properties of single GaAs nanowires with radial p-i-n junctions, using a nanoprobing setup. A uniaxial tensile strain of 3% resulted in an increase in photocurrent by more than a factor of 4 during NIR illumination. This effect is attributed to a decrease of 0.2 eV in nanowire bandgap energy, revealed by analysis of the current–voltage characteristics as a function of strain. This analysis also shows how other properties are affected by the strain, including the nanowire resistance. Furthermore, electron-beam-induced current maps show that the charge collection efficiency within the nanowire is unaffected by strain measured up to 0.9%.

Semiconductor nanowires possess unique electronic and optoelectronic properties, making them potential building blocks for a range of advanced devices, including nanoscale transistors,^1^ photovoltaics, and photodetectors.^2,3^ Especially, III–V nanowires with built-in p–i–n junctions are promising candidates for the next-generation solar cells with high efficiency and low cost. This is because the nanowire structures have several inherent advantages, such as direct band gaps, light-trapping ability,^4−6^ the possibility to grow on lattice mismatched substrates, high electron and hole mobilities, and the opportunity to decouple the optical and electrical thickness of solar cells.^7,8^ A power conversion efficiency (PCE) higher than ∼15% under 1 sun illumination has been reported in GaAs nanowire-array solar cells fabricated on Si substrates.^9^ InP nanowire-array solar cells have also demonstrated an efficiency that exceeds the ray optics limit and reaches as high as 16.7%.^10,11^ Though promising, these efficiencies are still far below the theoretical PCE limits of the nanowire solar cells.^12^ As a result, intensive research efforts have been focused on further enhancing the PCE of the III–V nanowire solar cells by engineering their charge separation, collection and light absorption properties.^13,14^ Surface passivation has been used to minimize surface recombination of photogenerated charge carriers.^15−18^ The effect of the positions and dimensions of the nanowires on light absorption in nanowire-array solar cells has been studied.^7,19,20^ Engineering the light absorption of the nanowires by coupling them to plasmonic nanoparticles has also been demonstrated.^21^ Moreover, since the intrinsic light absorption and electrical transport properties of the nanowires are largely determined by their electronic band structures, modification of the band structures could enable efficient tuning of the photovoltaic properties of the nanowire solar cells.

Mechanical strain has been shown to be an effective means to alter the band structures of semiconductor nanostructures. Direct investigations of the effect of mechanical strain on the band structures of III–V nanowires have been carried out. Band gap modification by uniaxial strain has been found in zinc blende GaAs nanowires,^22^ and uniaxial stress has been used to induce a direct-to-indirect band gap transition in wurtzite GaAs nanowires.^23^ Large modifications in the band gap of GaAs nanowires have been realized by lattice mismatch between nanowire core and shell in GaAs/InGaAs core/shell nanowires.^24^ Band shift in GaAs/GaP core/shell nanowires due to strain has also been reported.^25^ There are several examples in different types of semiconducting structures where strain has enabled a controlled modification of the properties. For instance, electron transport properties of Si nanowires have been greatly enhanced by externally applied strain;^26^ tensile strain has been used to tune the electro-optical properties of Ge nanowires.^27^ A piezoelectric effect in ZnO nanowires has been revealed,^28^ and uniaxial tensile strain has been used to modify the charge transport properties of individual InAs and GaAs nanowires.^29,30^ Despite these evident effects of strain on the band structure and properties of III–V nanowires, especially GaAs nanowires, the impact of mechanical strain on the performance of III–V nanowire solar cells has not been investigated and is not fully understood.

Strain may modify the p–n junctions in the nanowires due to the change in band structure, influencing the separation of the electron–hole pairs in the solar cells. Strain may also cause changes in mobilities and diffusion lengths of charge carriers in the nanowires, affecting charge collection. The strain-induced change in band structure can potentially alter light absorption as well, due to changes in band gap and dielectric function. It is thus of importance to study strain effects on light–nanowire interaction, charge transport, and photovoltaic properties of III–V nanowire solar cells for further developing and optimizing III–V nanowire solar cells via strain engineering. Studies of individual nanowires eliminate the averaging effect that measurements on nanowire-array solar cells suffer from and could allow a more accurate assessment of device physics in the strained solar cells. However, there are presently only a few studies on the characterization of single as-grown nanowire solar cells due to experimental challenges, such as device fabrication and realization of reliable contacts with the nanoscale solar cells.^31−37^ Strain-induced effects in single nanowire solar cells have not yet been experimentally examined.

In this study, we investigated the effect of mechanical strain on the photovoltaic properties of single GaAs nanowire solar cells. Using a scanning tunneling microscope–focused ion beam scanning electron microscope (STM-FIB-SEM) setup, tensile strain was applied to individual GaAs nanowires with radial p–i–n junctions. Current–voltage (*I*–*V*) measurements were performed on the nanowires under both dark conditions and under illumination by integrating light-emitting diodes (LEDs) in the FIB-SEM chamber. By quantitatively analyzing the *I*–*V* characteristics, the effect of strain on the band gap value, properties of Schottky barriers at the electrical contacts, nanowire resistance, as well as other electrical property parameters was unveiled. The short-circuit current during illumination with an LED with peak wavelength at 940 nm was found to increase with increasing strain. This effect is attributed to a reduction in band gap energy induced by the strain. Electron-beam-induced current (EBIC) measurements were used to examine the influence of uniaxial tensile strain on the charge collection process in the nanowire solar cells.

GaAs nanowires with lengths of 15–30 μm and diameters of 250–350 nm were grown on p-doped Si by self-catalyzed molecular beam epitaxy, for further details see ref. (33). A radial p–i–n junction within each nanowire was formed by adding a flux of beryllium (silicon) during the growth of the core (shell). The expected doping concentrations by comparison to planar growth are 3.5 × 10^19^ and 5 × 10^18^ cm^–3^ for the core and shell, respectively. An STM-SEM setup, described in detail in,^38^ was used to electrically contact individual, as-grown nanowires. All experiments were performed in an FEI Versa 3D FIB-SEM. In order to optimize the electrical contact between the STM-probe and the nanowires, the native oxide layer covering the surface of the nanowires was milled away at the contact area using the FIB. An acceleration voltage of 2 kV and a beam current of 27 pA were chosen to avoid damaging the nanowires. Subsequently, the STM-probe and the n-doped shell of the nanowires were attached together by electron beam induced Pt-deposition, using a gas injection system in the SEM chamber. The STM-probe was connected to an external circuit containing a voltage supply and a picoammeter, enabling *I*–*V* characteristic measurements. All the *I*–*V* measurements were performed with the electron beam turned off. The p-doped core of the nanowires was connected to the external circuit through the substrate, using conductive silver paint. The illumination of the nanowires was enabled by mounting an LED 1 cm away from the nanowire sample. The two different LEDs that were used were the 525 nm green 3 mm T-1 and the IRLED 940 nm, 3 mm from Würth Elektronik. The LEDs had a light intensity of 15 mW/sr and 30 mW/sr, respectively.

Individual as-grown GaAs nanowires with built-in p–i–n junctions were contacted and strained by retracting the STM-probe, as shown in Figure 1a. The *I*–*V* characteristics were measured for several individual nanowires as a function of tensile strain. A few examples of representative *I*–*V* characteristics and corresponding data-fitting are shown in Figure 1c,d. It is evident that the strain has a large impact on the *I*–*V* characteristics.

**Figure 1:**
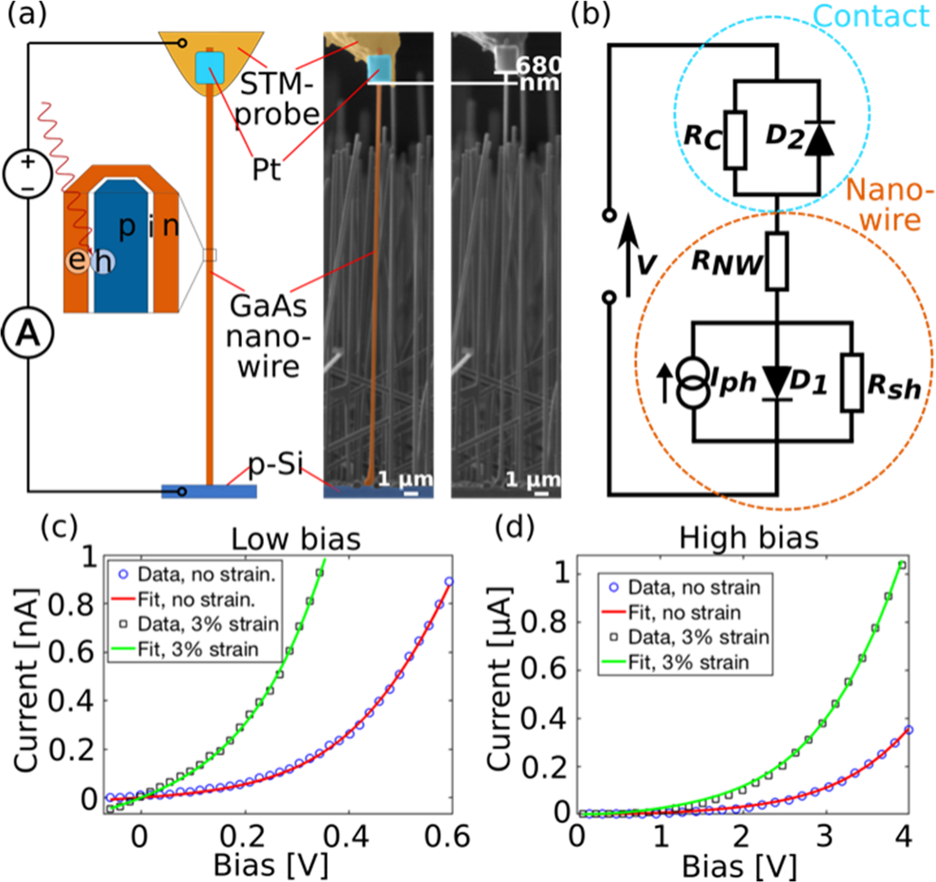
(a) The STM-probe is used inside a FIB-SEM to contact individual nanowires. The contact is stabilized by Pt-deposition. The cross-sectional view, in the schematic part of the figure, shows the p-i-n core–shell structure of the nanowire. When the nanowire is illuminated by the LED, electron–hole pairs are created and then separated by the built-in field in the depletion region of the p-i-n junction. An external circuit containing a power supply and a picoammeter enables *I*–*V* measurements. The SEM micrograph to the left shows a contacted nanowire in the relaxed state, using false colors to highlight the different parts of the setup. The SEM micrograph to the right shows the same nanowire at an elongation of 680 nm, corresponding to a strain level of around 3%. (b) Electrical circuit used to model the experimental system for quantitative analysis. (c, d) Dark *I*–*V* characteristics in the low and high bias regime, respectively, for a representative nanowire at two different strain levels, and the corresponding fits.

The quantitative effect of the strain on the electrical properties was evaluated. The experimental system was described by the model presented in Figure 1b. The nanowire is represented by a p–n diode (*D*_1_), a current generator, series resistance (*R*_NW_), and shunt resistance (*R*_Sh_). The contact between the STM-probe and the nanowire is represented by a Schottky diode (*D*_2_) and shunt resistance (*R*_C_). A more detailed description of the model is presented in Supporting Information section S1. By using this model to create fits to the experimental dark *I*–*V* curves, we have extracted the change in the p–n diode saturation current (*I*_s_), the Schottky barrier height (ϕ_b_) and the resistances *R*_NW_, *R*_Sh_, and *R*_C_ as a function of applied strain; see Figure 2. The nanowire strain was cycled in two subsequent series, returning to the relaxed state between and after the series. At each measurement point, the uniaxial tensile strain was calculated by dividing the elongation of the nanowire with its original length, all directly measurable from SEM images. Snapshots and videos showing the straining are provided in Supporting Information section S6 and Video S1, S2, S3, and S4. There is good agreement between the data for the different series in Figure 2. We can therefore conclude that the effect of strain on the electrical properties of the nanowire is reproducible and reversible.

**Figure 2:**
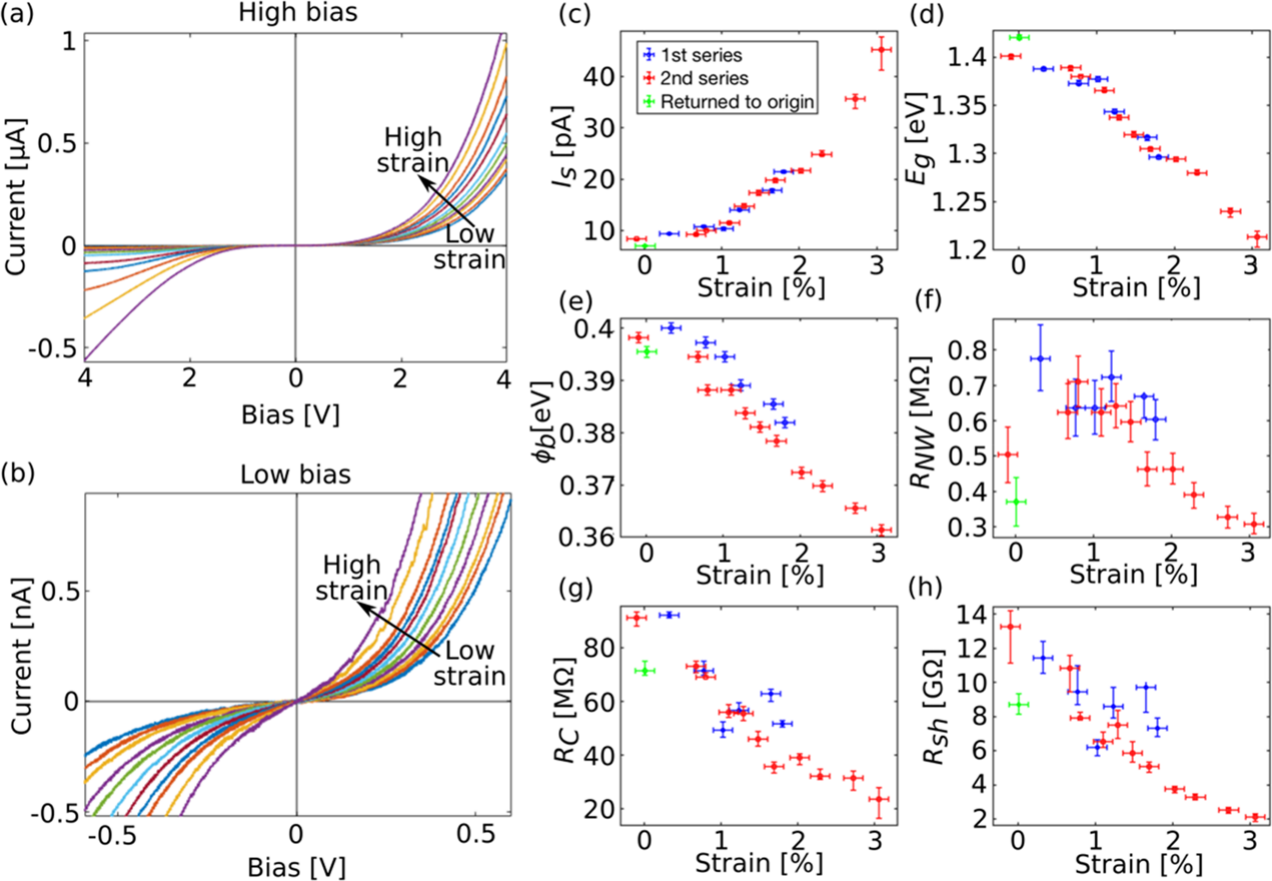
(a, b) Dark *I*–*V* characteristics of a single nanowire during the second straining series in the high and low bias regimes, respectively. (c–h) show *I*_S_, *E*_g_, ϕ_b_, *R*_NW_, *R*_sh_, and *R*_sh_ as a function of applied tensile strain, respectively. The values are extracted from the data-fitting. The vertical error bars correspond to the 99% confidence intervals obtained by the least-squares method. The horizontal error bars are based on an estimation of the accuracy in determining the elongation of the nanowire from the SEM images.

The band gap energy (*E*_g_) of the nanowire was extracted using the expression^39^
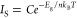
1where *n* is the diode ideality factor, *k*_B_ Boltzmann’s constant and *T* the temperature. *C* was determined using the tabulated band gap energy for GaAs and the extracted value of *I*_S_ at zero applied strain. In Figure 2d, we see that *E*_g_ decreases approximately linearly with increasing strain. At a strain level of about 3% the change in *E*_g_ is 0.2 eV. This is in accordance with other studies on similar nanowires, where photoluminescence^22,24^ and electron energy loss spectroscopy^40^ have been used to determine *E*_g_ as a function of strain. In Figure 2e, it can be seen that ϕ_b_ decreases with around 40 meV at 3% strain. A possible explanation for this is that for a metal–GaAs contact the metal Fermi level is usually pinned to an energy level *E*_p_ within the band gap.^41^ Then ϕ_b_ will depend on the distance between the bottom of the conduction band and *E*_p_. Since *E*_g_ of the nanowire decreases with increasing strain, the conduction band is most likely shifted downward. Assuming that *E*_p_ is constant, this results in a lower ϕ_b_.

*R*_NW_ is a combination of the resistance of the n-doped shell, *R*_shell_, and the p-doped core, *R*_core_. Considering the electron and hole mobilities of GaAs^42^ and the doping concentrations of the core and shell, *R*_core_ is expected to be around 5 times higher than *R*_shell_. *R*_core_ is therefore regarded as the dominating factor that determines the value of *R*_NW_. In Figure 2f, we see that *R*_NW_ increases initially with increasing strain, but at higher strain, it decreases. This effect is believed to be due to the splitting of the heavy and light hole bands induced by the strain^43^ and is described in more detail in ref (40). *R*_C_ decreases with the strain (Figure 2g); this could possibly be because the mechanical contact between the nanowire and the deposited Pt improves when there is a force acting on it. *R*_sh_ decreases as well (Figure 2h); the reason for this is unclear. Repeated straining measurements during dark conditions on a different nanowire are presented in Supporting Information section S2.

*I*–*V* characteristics as a function of strain were also measured under two kinds of illumination; see Figure 3b. Figure 3a shows the wavelength spectra of two different LEDs, one green and one infrared, that were used to illuminate the nanowires. The solar spectrum is included in order to illustrate the additional part of the sunlight that can be absorbed by the nanowire when it is strained. Figure 3c shows the short-circuit current, *I*_SC_, as a function of strain for both NIR and green LED illumination. The measurements under different illumination were performed on different nanowires. During NIR illumination, *I*_SC_ increases in an S-shaped fashion with the highest increase-rate at approximately 1.3% strain. The curve flattens out at 2–3% strain. In Figure 2b, we see that the calculated *E*_g_ is around 1.33 eV at 1.3% strain. This corresponds to a photon wavelength of 930 nm, which is close to the peak wavelength of the NIR LED, meaning that a small reduction in *E*_g_ results in a large additional absorption. At 2–3% strain, the calculated *E*_g_ corresponds to a wavelength of 970–1030 nm. In this range, the intensity of the LED is quickly decreasing, so a further reduction in *E*_g_ results in less and less additional absorption. The increase in *I*_SC_ is thus consistent with the change in *E*_g_ as a function of strain extracted from *I*–*V* characteristics. Furthermore, if the measured increase in *I*_SC_ would have been due to changes other than the band gap variation of the nanowire, then it would have occurred regardless of the wavelength of the illumination. For green illumination though, *I*_SC_ is large already at zero strain and does not increase. The data for the green illumination are not provided for strain higher than 1%. The reason is that the nanowire detached from the substrate before fracture. However, the relatively high and stable *I*_SC_ under strain for the green LED illumination confirms that the increase in *I*_SC_ during NIR illumination was due to a change in band gap.

**Figure 3:**
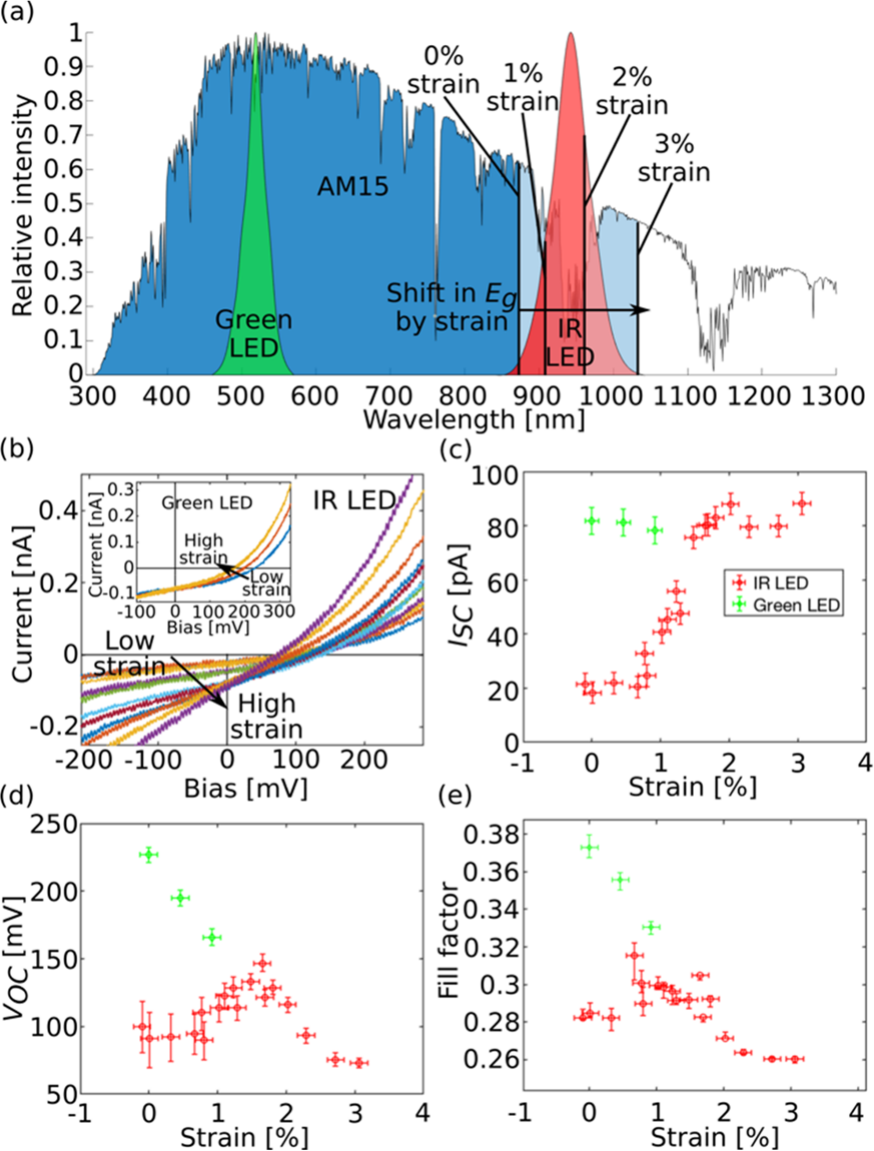
(a) Wavelength spectrum of the two LEDs that were used to illuminate the nanowires, and the AM15 solar spectrum. The intensity of each individual spectrum is normalized and plotted along the *y*-axis. The wavelengths corresponding to the calculated *E*_g_ at three different strain levels have been indicated. (b) *I*–*V* characteristics in the low bias regime under illumination by the NIR LED and the green LED (inset). The measurements with the green LED and the NIR LED were performed on two different nanowires. (c–e) *I*_SC_, *V*_OC_, and fill factor, respectively, for the nanowires during the two types of illumination (green and NIR LED) as a function of applied strain.

The open-circuit voltage (*V*_OC_) of a solar cell can be expressed as
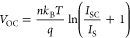
2if all parasitic resistances are neglected. We know that *I*_S_ increases with strain, and for the green LED, *I*_SC_ is almost unchanged. From eq 2 we therefore expect that the *V*_OC_ decreases when strain is applied. This is confirmed by the measurements, see Figure 3d. For illumination with the NIR LED though, there is an increase in *I*_SC_, causing *V*_OC_ to increase initially. In contrast, at high strain levels, *I*_SC_ no longer increases, and *V*_OC_ starts to decrease. Additionally, *R*_sh_ is reduced significantly at this high strain, which can have a detrimental effect on *V*_OC_. The fill factor increases slightly at low strain during NIR illumination, but at higher strain levels and during green illumination, it decreases with increasing strain (see Figure 3e). This decrease is most likely due to the reduction in *R*_sh_. Repeated straining measurements during NIR LED illumination on two different nanowires are presented in Supporting Information sections S3 and S4.

Electron-beam-induced current (EBIC) maps can provide information about the spatial charge carrier collection efficiency within individual nanowires. Figure 4a shows an example of an EBIC map and the corresponding SEM micrograph. There is a high EBIC signal along the whole nanowire, except where the Pt-deposition blocks the incoming electrons. This confirms the existence and the radial geometry of the p–i–n junction. The radial geometry ensures that the distance the generated charge carriers need to diffuse to reach the junction always is smaller than the radius of the nanowire. The amount of charge carriers that recombine before they reach the junction should therefore be the same regardless of where along the nanowire the electron beam is placed. This is opposed to the axial geometry where the diffusion distance varies along the nanowire. There is a continuous increase in the EBIC signal going from top to bottom in the nanowire. The probable cause of this is that when the separated charge carriers are transported toward the contacts some of them are redistributed into the junction and recombined because of the finite resistance within the nanowire.^44^ The higher the resistance, the more recombination that will occur. As mentioned earlier, *R*_core_ is estimated to be much larger than *R*_shell_. Thus, the shorter the distance between the region where the charges are created and the core contact, the lesser the recombination and the larger the EBIC. In Figure 4b, the EBIC line profiles along the center of the wire at different strain levels are shown. Here we see that when the wire is strained the EBIC signal is not affected. One prerequisite for this is that the deformation of the wire is elastic so that no additional defects, acting as recombination centers, appears in the nanowire during the straining. The fact that the *I*–*V* characteristics of the nanowires go back to the original when the strain is relaxed implicates that the deformation is elastic. In a previous study,^40^ in situ TEM strain mapping and linear stress–strain curves also showed elastic deformation all the way up to fracture for similar GaAs nanowires. This means that the charge carrier collection efficiency is not impaired when the nanowire is strained. A way to utilize the effect of strain could therefore be to tune the band gap, enabling optimal use of the incoming light. The optimal *E*_g_ for a single-band gap solar cell under AM15 illumination is 1.34 eV.^45^ According to our results, this value of *E*_g_ can be reached in a GaAs nanowire solar cell by applying approximately 1.3% tensile strain to the nanowires. Of course, there is only a little room for improvement in this way since the band gap of GaAs is already close to the optimal value. However, if the nanowires can be strained inhomogeneously, then a larger increase in efficiency could potentially be obtained. For example, a strain gradient going from high strain at the bottom of a nanowire to low strain at the top would result in a continuous decrease in *E*_g_ from top to bottom. This would create the same type of beneficial effect as in a multijunction solar cell, without the need for fabrication of complicated heterostructures.

**Figure 4:**
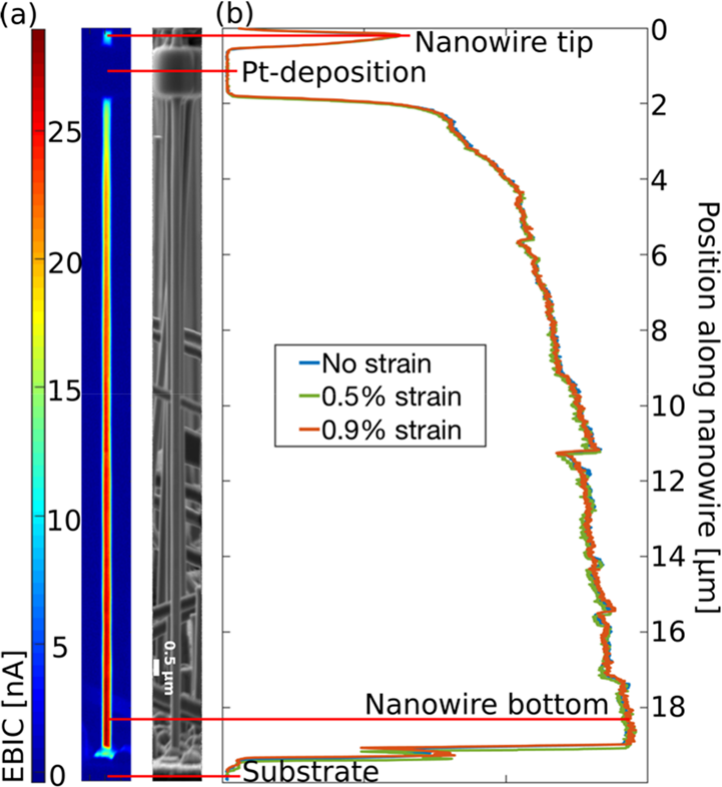
(a) EBIC map and corresponding SEM micrograph for the unstrained nanowire. (b) EBIC line profile along the center of the nanowire at three different strain levels.

We have investigated the effect of uniaxial tensile strain on the electronic and photovoltaic properties of individual GaAs nanowires with radial p–i–n junctions. By fitting the experimentally measured *I*–*V* characteristics to a theoretical model of the nanowire–contact system, the variation of parameters such as *E*_g_ and *R*_NW_ as a function of strain were extracted. *E*_g_ was found to decrease by 0.2 eV at 3% strain. As a result of the decrease in *E*_g_, the maximum wavelength of absorption by the nanowire is shifted from around 870 to 1030 nm. We have shown this experimentally by straining the nanowire while illuminating it with an LED centered at 940 nm. The *J*_SC_ increased gradually from 39 ± 10 to 173 ± 23 mA/cm^2^ as the strain increased. We believe our results can be utilized in making nanowire solar cells more efficient, especially if the nanowires can be strained inhomogeneously, effectively creating a distributed multijunction solar cell. Our findings may also be applicable to nanowire-based photodetectors where an ability to tune the wavelength sensitivity is advantageous.

## Supporting Information Available

Theoretical model for analysis of dark *I*–*V* characteristics; dark *I*–*V* measurements on Nanowire 3; illuminated *I*–*V* measurements on Nanowire 3; dark *I*–*V* measurements on unmilled Nanowire 4; illuminated *I*–*V* measurements on unmilled Nanowire 4; STM-SEM sample holder (PDF)
Videos showing the straining of Nanowires 1–4 (MOV, MOV, MOV, MOV)

## Supplementary Material

nl1c02468_si_001.pdf

nl1c02468_si_002.mov

nl1c02468_si_003.mov

nl1c02468_si_004.mov

nl1c02468_si_005.mov
