# Supplementary material for: Enhancing the NIR Photocurrent in Single GaAs Nanowires with Radial p-i-n Junctions by Uniaxial Strain
Source: Nano Lett. 2021 Oct 27;21(21):9038–43. doi: 10.1021/acs.nanolett.1c02468 (PMC8587900; doi:10.1021/acs.nanolett.1c02468)
Supplement: Supplementary file 1 — nl1c02468_si_001.pdf [file nl1c02468_si_001.pdf]

Supporting information

Enhancing the NIR photocurrent in single GaAs nanowires with radial p-i-n junctions by uniaxial strain

Jonatan Holmér[1], Lunjie Zeng[1*], Thomas Kanne[2], Peter Krogstrup[2], Jesper Nygård[2], Eva Olsson[1*]

1 Department of Physics, Chalmers University of Technology, 412 96 Gothenburg, Sweden

2 Center for Quantum Devices, Niels Bohr Institute, University of Copenhagen, Universitetsparken 5, 2100 Copenhagen, Denmark

*Corresponding authors: Lunjie Zeng (lunjie@chalmers.se) and Eva Olsson (eva.olsson@chalmers.se).

Supporting information

S1. Theoretical model for analysis of dark I-V characteristics

The electrical circuit that was used to model the nanowire-contact system is shown in Figure 1. In this configuration, a positive applied bias corresponds to a forward biased p-n diode and a reversed biased Schottky diode. The current through the p-n diode, I_{D1}, is described by the single diode model for a non-ideal solar cell [1],

$$I_{D1} = I_S\left(e^{qV_{D1}/nk_BT} - 1\right), \tag{1}$$

where V_{D1} is the voltage drop over D_1, I_S is the diode saturation current, n is the diode ideality factor, q is the elementary charge, k_B is Boltzmann's constant and T is the temperature. Applying Kirchhoff's law to the model circuit, we then have an expression for the total current, I_{tot},

$$I_{tot} = I_{D1} + \frac{V_{D1}}{R_{Sh}} - I_{ph} = I_{D2} + \frac{V_{D_2}}{R_C} = \frac{V_{R_{NW}}}{R_{NW}}, \tag{2}$$

and for the applied bias, V,

$$V = V_{D1} + V_{D2} + V_{R_{NW}}. \tag{3}$$

where V_{D1}, V_{D2} and $V_{R_{NW}}$ are the voltage drops over the D_1, D_2 and R_{NW}, respectively and I_{ph} is the photogenerated current. In the low bias regime, the current through the reversed biased Schottky diode, I_{D2}, is negligibly small and the contact can be regarded as only a resistance, R_C. Combining (1), (2) and (3) we get the following expression for I_{tot} as a function of applied bias, in the low bias regime:

$$I_{tot}(V) = \frac{I_{ph} + I_S - {}^{V}/_{R_{sh}}}{1 + {}^{(R_C + R_{NW})}/_{R_{sh}}} - \frac{nV_T}{R_C + R_{NW}} W \left(\frac{I_S(R_C + R_{NW})}{nV_T\left(1 + {}^{(R_C + R_{NW})}/_{R_{sh}}\right)} exp\left(\frac{V}{nV_T}\left(1 - \frac{R_C + R_{NW}}{R_C + R_{NW} + R_{sh}}\right) + \frac{(I_{ph} + I_S)(R_C + R_{NW})}{nV_T\left(1 + {}^{R_C + R_{NW}}/_{R_{sh}}\right)} \right)\right).$$

(4)

Here $V_T = \frac{k_B T}{q}$ and W is the Lambert W function. In the high bias regime, the voltage drop over the Schottky diode will be sufficient for thermionic field emission to occur, resulting in a significant current through the contact even though the Schottky barrier is reversely biased. According to thermionic field emission theory [2,3], the current through the diode is

$$I_{D2} = I_{sb}(V_{D2}, \phi_b) \times exp\left[V_{D2}\left(\frac{q}{k_B T} - \frac{1}{E_0}\right)\right], \tag{5}$$

where ϕ_b is the Schottky barrier height and $E_0 = E_{00} coth\left(\frac{qE_{00}}{k_B T}\right)$ with $E_{00} = \frac{\hbar}{2}\left[\frac{N_d}{m_n^* \epsilon}\right]^{1/2}$. N_d is the doping concentration of the semiconductor, m_n^* is the effective electron mass and ϵ is the permittivity of the nanowire. The saturation current for the Schottky diode, I_{sb}, can be expressed as:

$$I_{sb} = \frac{SA^* T(\pi q E_{00})^{1/2}}{k_B} exp\left(-\frac{\phi_b}{qE_0}\right) \times \left\{q(V_{D2} - \xi) + \frac{\phi_b}{cosh^2\left({}^{qE_{00}}/_{k_B T}\right)}\right\}^{1/2}. \tag{6}$$

Here S is the contact area, A^* the effective Richardson's constant and ξ is the difference between the Fermi energy and the bottom of the conduction band. Solving (2), (3) and (5) numerically, it is possible to calculate the total current as a function of applied voltage also in the high bias regime.

S2. Dark I-V measurements on Nanowire 3

A third nanowire was contacted with the STM-probe in the same manner as described in the main article. Dark I-V measurements were performed at different strain levels, and the parameters I_s, E_g, ϕ_b, R_{se}, R_{sh} and R_C were extracted by data-fitting. When going from low to high strain, all the parameters follow the same trends as Nanowire 1 described in Figure 2 in the main article.

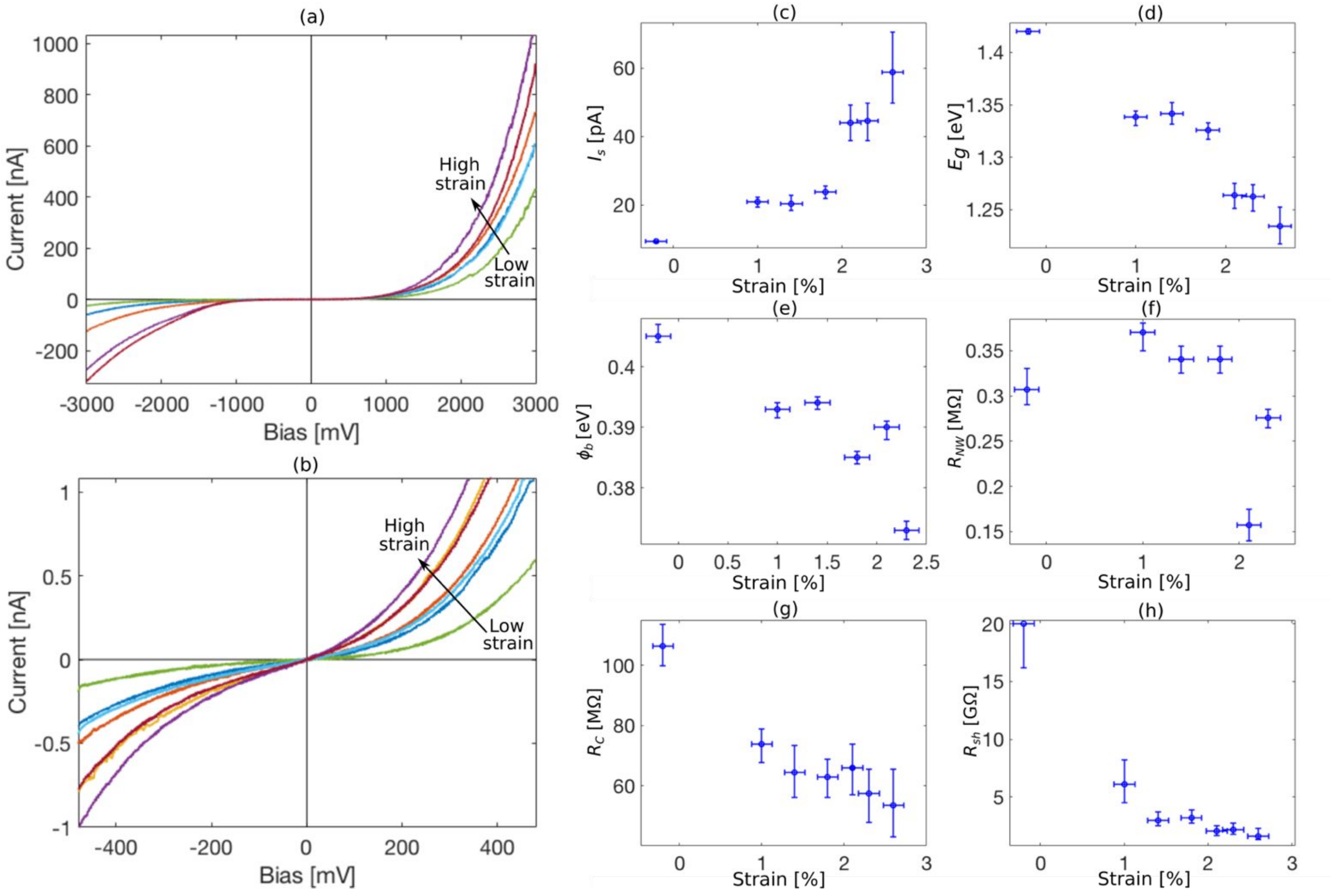

Figure S2: (a-b) Dark I-V characteristics of Nanowire 3 at different strain levels in the high and low bias regimes, respectively. (c-h) shows I_S, E_g, ϕ_b, R_{NW}, R_{sh} and R_{sh} as a function of applied tensile strain, respectively. The values are extracted from the data-fitting.

S3. Illuminated I-V measurements on Nanowire 3

Nanowire 3 was also illuminated by the NIR LED during the straining. The I_{SC} increases with increasing strain, but not as much as in Nanowire 1. One explanation for this is that the shape of the STM-probe varied between the measurements, since it is produced by mechanically cutting a gold wire with a pair of scissors. The STM-probe may partly block the incoming LED light, and the area of the blocked part may thus change whenever a new STM-probe is used. The flattening out of the curve is not observed because the maximum strain reached is lower than for Nanowire 1. Both in Nanowire 1 and Nanowire 3, the I_{SC} is nonzero even without any applied strain. Assuming that the band gap energy of the unstrained nanowire is close to the tabulated value for bulk GaAs (1.42 eV), almost no part of the LED spectrum can be absorbed in the nanowire when no strain is applied. The nonzero I_{SC} is therefore assumed to originate from electron-hole pairs generated in the Si substrate, which diffuse into the nanowires and are separated by the p-i-n junction. The V_{OC} of Nanowire 3 decreases as a function of strain. The reason that there is no increase at moderate strain levels is probably because the increase in I_{SC} is smaller than for Nanowire 1. The fill factor also decreases with increasing strain, which is most likely a consequence of the decrease in R_{sh}.

Supporting information

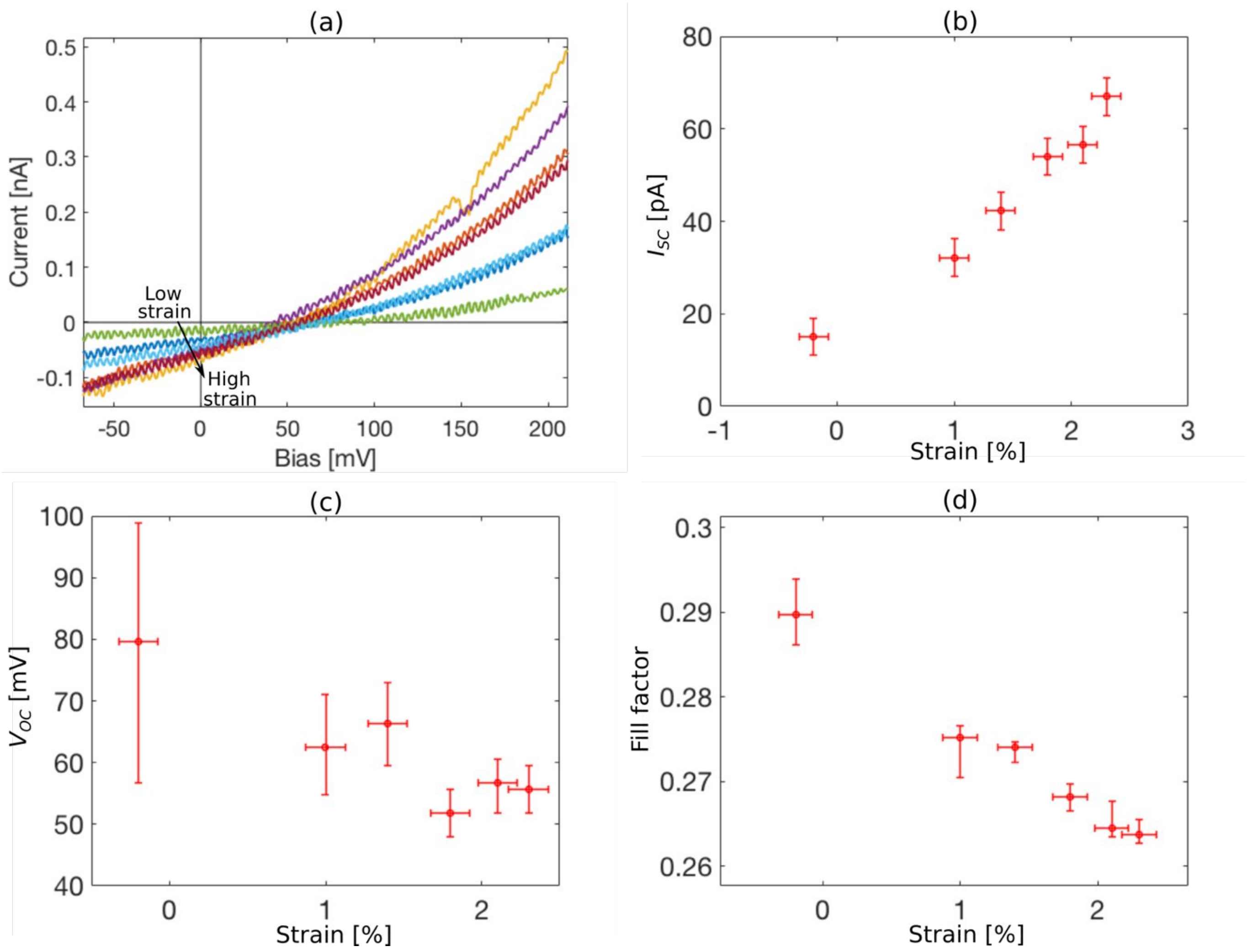

Figure S3: (a) Illuminated I-V characteristics of Nanowire 3 in the low bias regime at different strain levels. (b-d) I_{SC}, V_{OC} and fill factor for Nanowire 3 as a function of strain.

S4. Dark I-V measurements on unmilled Nanowire 4

I-V measurements at different strain levels were performed on a fourth nanowire, Nanowire 4. The difference here is that this nanowire was not milled with the FIB before depositing the Pt creating the contact between the nanowire and the STM-probe. The native oxide layer covering the surface of the nanowire was therefore still intact, acting as a thin insulating layer, making the electrical contact worse. This can be seen by noting that a higher bias was needed to reach the same current compared to the milled Nanowire 1, see Figure 2 (a) and (b). Otherwise, the I-V characteristics of the milled Nanowire 1 and the unmilled Nanowire 4 were very similar. In particular, at low strain there was a distinct current rectification in both wires, caused by the p-i-n junction. The fact that there was a current rectification behavior in the milled Nanowire 1 shows that the FIB milling did not harm the p-i-n junction in the nanowire.

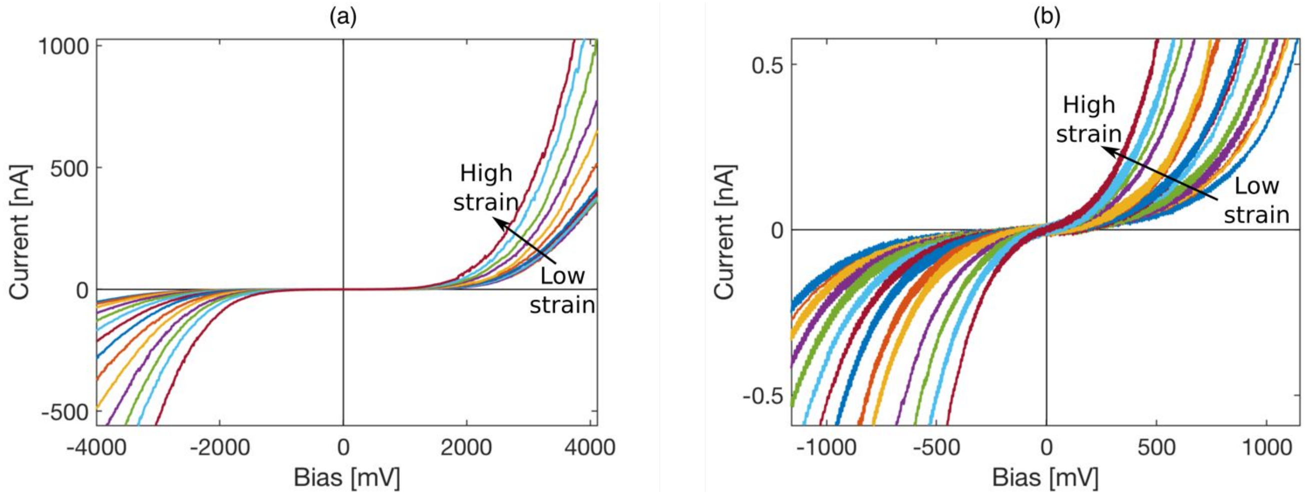

Figure S4: (a-b) Dark I-V characteristics of Nanowire 4 at different strain levels in the high and low bias regimes, respectively.

S5. Illuminated I-V measurements on unmilled Nanowire 4

I-V measurements during NIR LED illumination at different strain levels were also performed on the unmilled Nanowire 4. In Nanowire 4 the I_{SC}, V_{OC} and fill factor followed the same trends as in Nanowire 1 as the strain was increased, which shows that the FIB-milling of Nanowire 1 did not influence the strain effect of its photovoltaic behavior. The I_{SC} and V_{OC} of Nanowire 4 reached higher values than Nanowire 1, which is again likely due to the fact that a differently shaped STM-probe was used, in this case assumingly blocking a lesser part of the light. The noise that is visible in the I-V curves is the 50 Hz noise from the environment. This noise was present in all our measurements and varied in the range of 5-15 pA between the different experimental sessions. The noise does have an effect on the accuracy of the measured parameters I_{SC}, V_{OC} and fill factor. However, it does not affect the conclusions drawn about the strain-induced effects of the electrical and photovoltaic properties of the nanowires, since the shifts in the I-V curves are of a much larger magnitude than the noise.

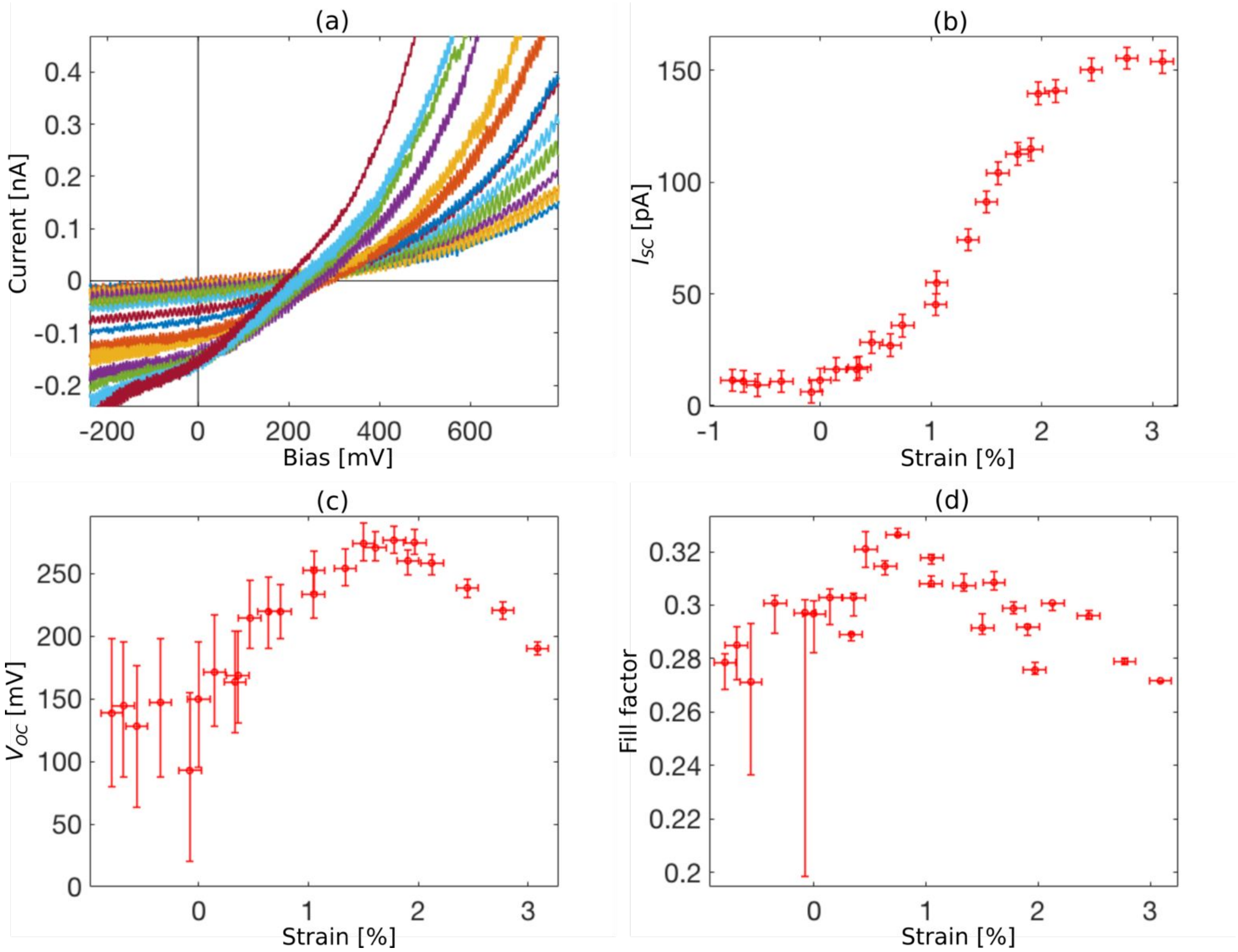

Figure S5: (a) Illuminated I-V characteristics of Nanowire 4 in the low bias regime at different strain levels. (b-d) I_{SC}, V_{OC} and fill factor for Nanowire 4 as a function of strain.

S6. Videos showing the straining of Nanowire 1-4

The SEM micrographs taken at all the different strain levels for Nanowire 1-4 have been merged into four separate videos. In the videos it can be seen that while the STM-probe is retracted, it also moves irregularly in the lateral directions, giving rise to a small shear component in the strain. However, the maximum lateral movement observed was on the order of 1 µm. The ratio between the shear and the uniaxial strain was estimated as the ratio between the lateral movement and the length of the nanowire. With this estimation,

the maximum shear strain was only 4 % of the uniaxial strain, and most of the time it was much less than that. The high reproducibility of the measurements, both between different nanowires and between different straining series of individual nanowires, further implicates that the uniaxial strain was dominating the shear strain.

Supporting information

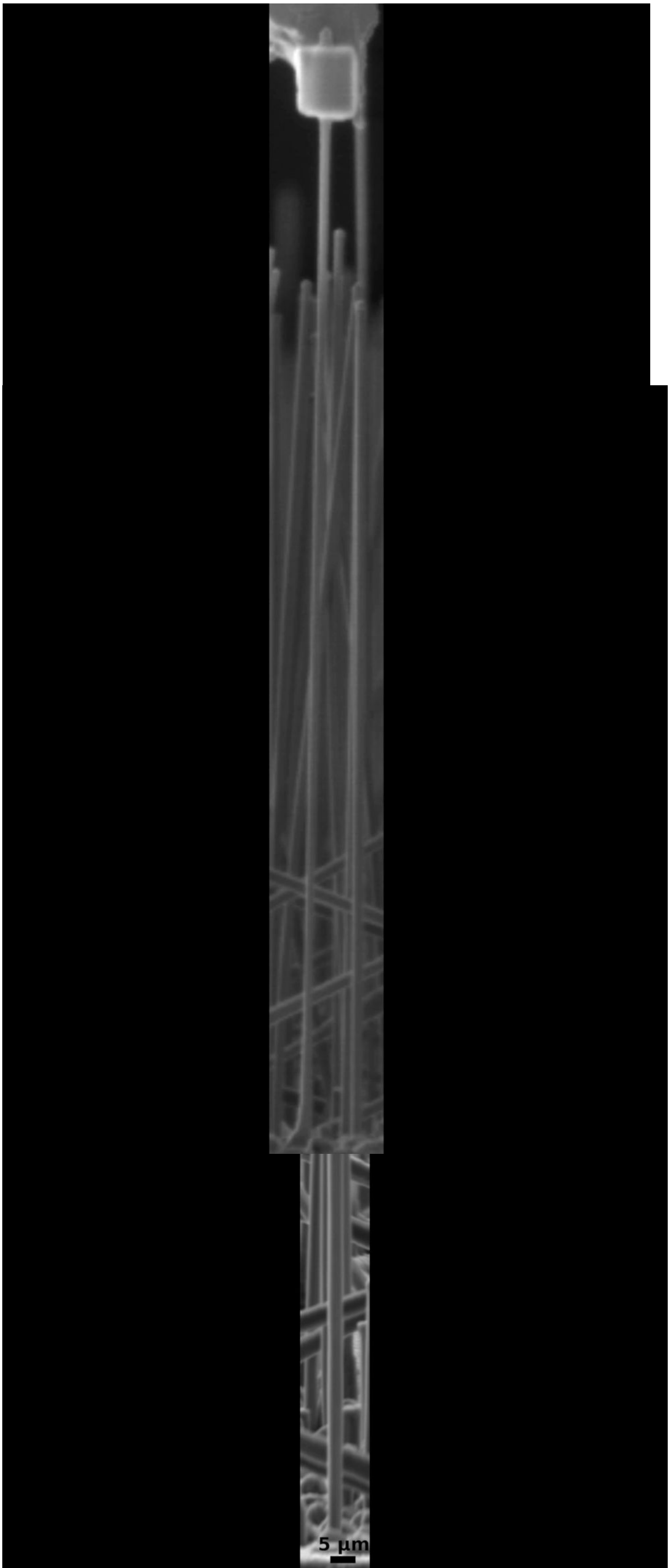

Video S1: Straining video of Nanowire 1

Supporting information

Video S2: Straining video of Nanowire 2. This is the nanowire that the green LED measurements and the EBIC maps are based on.

Supporting information

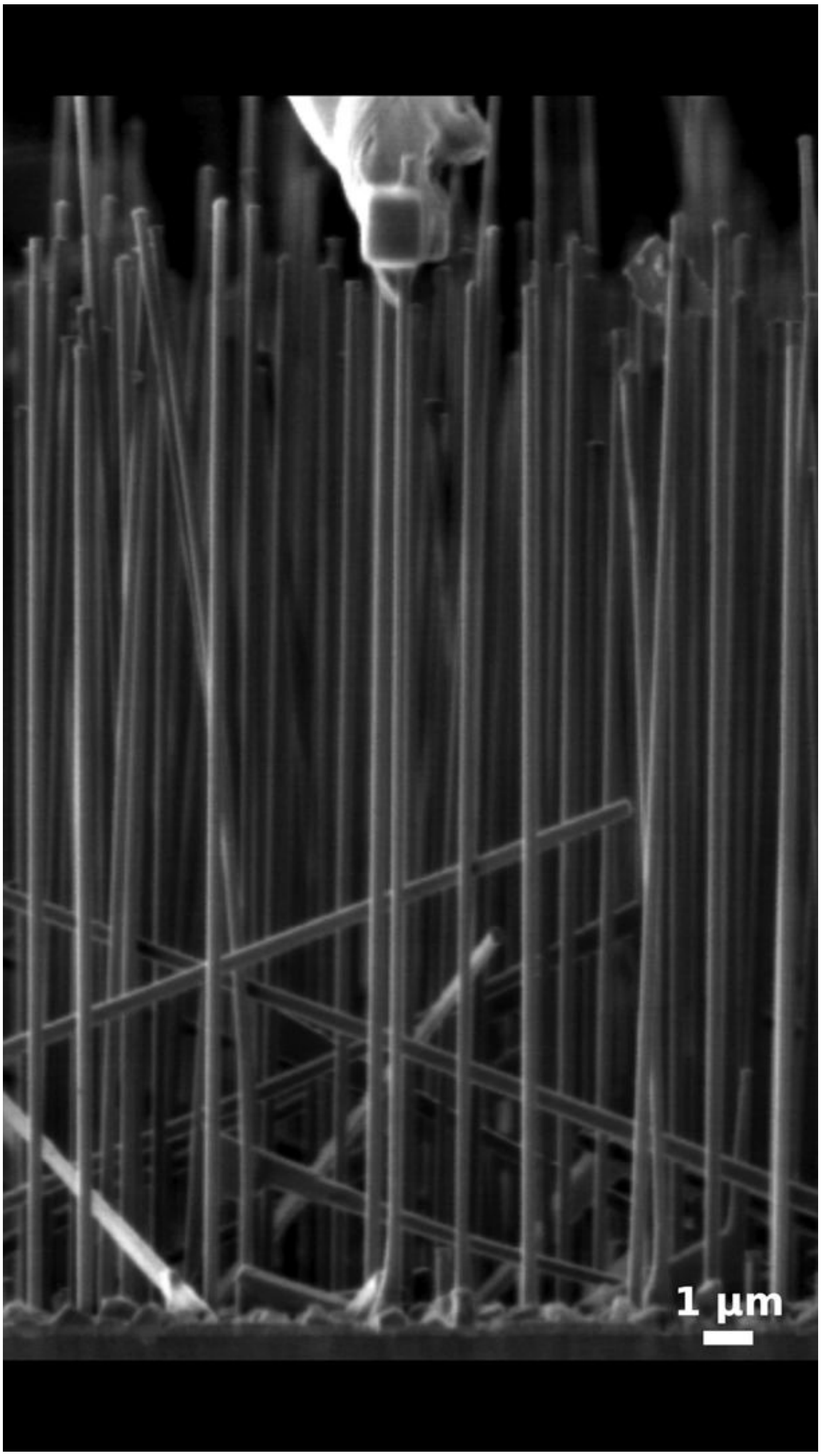

Video S3: Straining video of Nanowire 3.

Supporting information

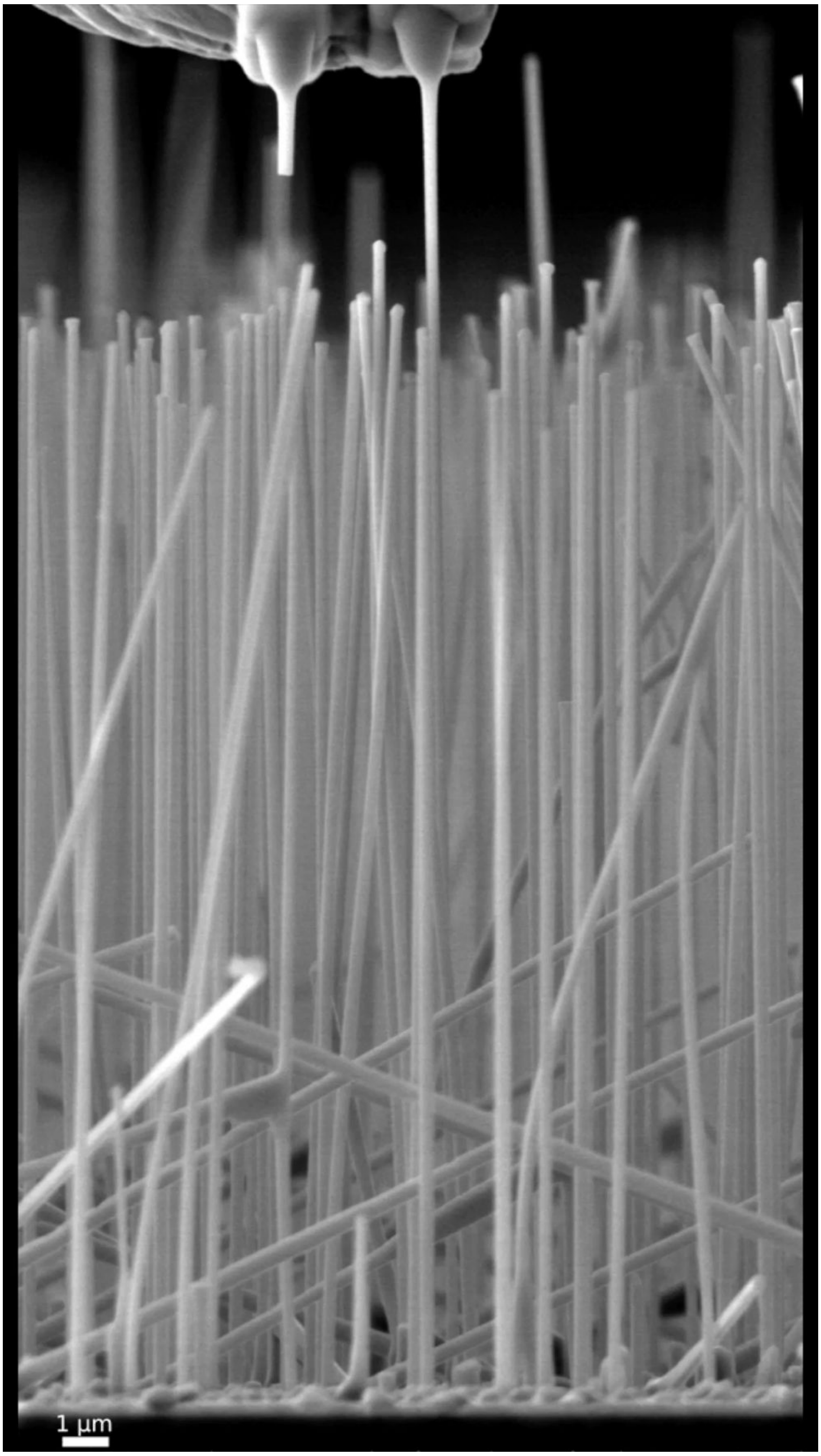

Video S4: Straining video of Nanowire 4.

S7. STM-SEM sample holder

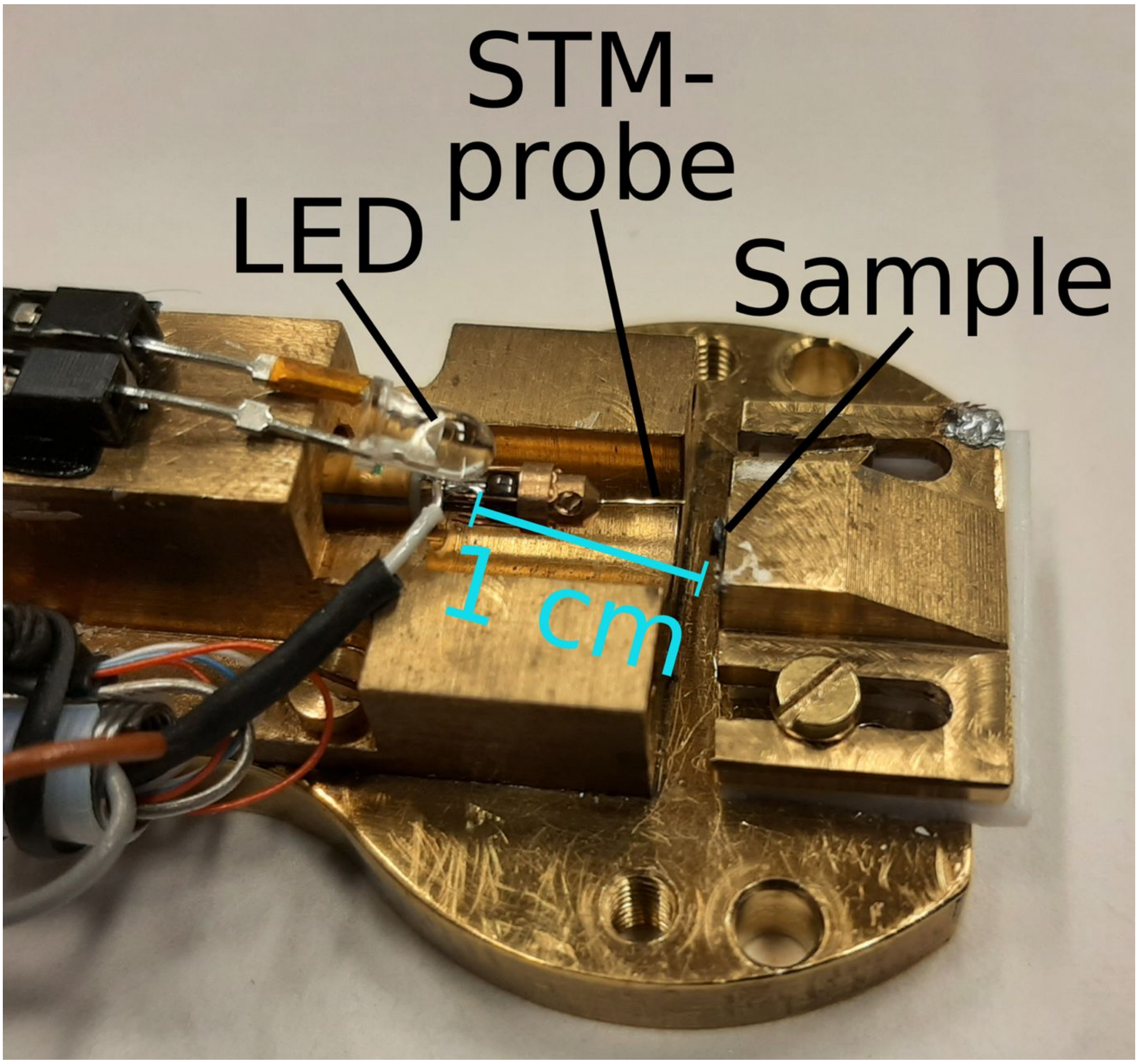

Figure S7: A photograph showing the STM-SEM sample holder. The STM-probe that was used to contact the nanowires, the nanowire sample and the LED that was used to illuminate the sample are highlighted in the photograph. The distance between the LED and the sample was 1 cm.

References

[1] R. Abbassi, A. Abbassi, M. Jemli, and S. Chebbi, *Identification of Unknown Parameters of Solar Cell Models: A Comprehensive Overview of Available Approaches*, Renew. Sustain. Energy Rev. **90**, 453 (2018).

[2] Z. Zhang, K. Yao, Y. Liu, C. Jin, X. Liang, Q. Chen, and L. M. Peng, *Quantitative Analysis of Current-Voltage Characteristics of Semiconducting Nanowires: Decoupling of Contact Effects*, Adv. Funct. Mater. **17**, 2478 (2007).

[3] F. A. Padovani and R. Stratton, *Field and Thermionic-Field Emission in Schottky Barriers*, Solid State Electron. **9**, 695 (1966).
